# Role of Secreted Frizzled-Related Protein 1 and Tumor Necrosis Factor-*α* (TNF-*α*) in Bone Loss of Patients with Rheumatoid Arthritis

**DOI:** 10.1155/2020/9149762

**Published:** 2020-03-01

**Authors:** Andi Raga Ginting, Rudy Hidayat, Sumariyono Sumariyono, Sukamto Koesnoe

**Affiliations:** ^1^Division of Rheumatology, Department of Internal Medicine, Dr. Cipto Mangunkusumo General Hospital, Faculty of Medicine Universitas Indonesia, Jakarta 10430, Indonesia; ^2^Department of Internal Medicine, Dr. Cipto Mangunkusumo General Hospital, Faculty of Medicine Universitas Indonesia, Jakarta 10430, Indonesia

## Abstract

Bone loss is one of the emerging extra-articular manifestations of rheumatoid arthritis. TNF-*α* is the main inflammatory cytokine that can directly increase bone resorption. However, its role in bone formation is still unknown, especially related to secreted frizzled-related protein 1 (SFRP-1), an osteoblast inhibitor. This study examines the correlation between TNF-*α* and SFRP-1, with a bone turn over marker (CTX and P1NP). This is a cross-sectional study with 38 subjects of premenopausal female patients with RA. This study found that 60.6% of the patients were in remission or low disease activity. The median of TNF-*α* was 10.6 pg/mL, mean of SFRP-1 was 9.29 ng/mL, mean of CTX was 2.74 ng/mL, and the median of P1NP was 34.04 pg/ml. There is positive correlation between TNF-*α* and P1NP (*r* = 0.363, *p* = 0.026), also between SFRP-1 and P1NP (*r* = 0.341; *p* = 0.036). A low level of TNF-𝛼, high level of SFRP-1, high level of CTX, and low level of P1NP in this study indicate a high bone turn over process, with dominant resorption activity in premenopausal female patients with RA.

## 1. Introduction

Rheumatoid arthritis (AR) is a chronic inflammatory systemic disease, which may cause progressive damage to bone and cartilage [1]. The prevalence and incidence of RA vary between one population to another. The prevalence of RA is around 1% in adult Caucasians in the United States and some European regions. Meanwhile, it is approximately 0.28% in China, 1.7% in Japan, and 0.75% in India. In Indonesia, based on the epidemiological survey results in Bandungan, Central Java, the prevalence of RA is 0.3% [2, 3].

The general features and characteristics of RA are joint inflammation that is often associated with erosion in synovial tissue that proliferates (pannus), and it invades the cortical, subchondral, and trabecular bones [4]. This articular erosion is clinically meaningful since it may cause systemic loss of bone mass [5]. It is proven by several studies, which show that RA patients have a lower bone mineral density (BMD) compared to healthy patients in the control group [6, 7]. Even, BMD results show that the majority of premenopausal female patients with RA are affected by osteopenia [6, 8]. Meta-analysis by Xue et al. [9] concluded that there is an increase in the incidence of fracture in RA patients with a relative risk of 2.25 compared to non-RA patients.

Systemic inflammation in patients with RA induced proinflammatory cytokine production, especially TNF-*α*, which will increase osteoclast differentiation and activity and reduce osteoblast differentiation and function by influencing Wingless and Int-1 (Wnt) signaling pathways, which is one of the osteogenic pathways [10]. Eventually, it will lead to bone erosion [4, 5]. Aggressive treatment of inflammation in RA may suppress bone resorption processes by osteoclasts, but improvement in bone erosion mediated by osteoblast only occurs in well-controlled clinical conditions [11]. The observation shows that in patients who do not achieve improvement in bone erosion, subclinical inflammation in the joints continues and suppresses osteoblast activity to repair erosion [12].

Regulation of Wnt signaling is controlled by some secreted antagonists, including members of secreted frizzled-related protein (SFRP). SFRP is a specific glycoprotein that binds to the Wnt ligand to prevent interaction with the receptor complex, so that the activation of the Wnt signaling pathway does not occur, which results in inhibition of osteoblast proliferation and differentiation. Several studies on the RA rat models found an increase in the expression of SFRP-1 in the synovial tissue compared to that of nonarthritis tissue [13, 14]. Also, there is an increase in the expression of SFRP-1 gene in the synovial tissue of RA human, especially in populations that are rich in fibroblast-like cells [15].

The process of bone metabolism can be assessed by the presence of biochemical markers [16, 17]. The *International Osteoporosis Foundation* (IOF) and the *International Federation of Clinical Chemistry and Laboratory Medicine* (IFCC) have proposed the *carboxy-terminal cross-linking telopeptide of type I collagen* (CTX) serum, which is a degradation product of osteoclast or from collagen degradation, to be used as a marker of bone resorption and *Procollagen Type 1 N Propeptide* (P1NP) serum, which is produced by osteoblastic cells or from procollagen metabolism, to be used as a marker of bone formation. The two markers are referenced in international observational studies and interventions [18].

This study is aimed at conducting an in-depth examination of the mechanisms of inflammatory bone loss in RA patients that causes an imbalance of bone metabolism by osteoclasts and osteoblasts. Since TNF-*α* is a proinflammatory cytokine that mainly influences osteoclastogenesis and SFRP-1 is a specific inhibitor of Wnt signaling that inhibits osteoblastogenesis, thus the correlation between TNF-*α* and SFRP-1 and bone turnover markers in premenopausal female patients with RA is examined. Premenopausal women were selected as the population of the study to eliminate confounding due to the effects of postmenopausal estrogen deficiency. So far, there is no study that directly correlates TNF-*α* and SFRP-1 with CTX and P1NP, so that this study is expected to provide a better understanding of bone metabolism in patients with rheumatoid arthritis.

## 2. Materials and Methods

This study used a cross-sectional design and was conducted at the Rheumatology Clinic of Cipto Mangunkusumo Hospital (RSCM) in April–May 2019. The research sample was premenopausal female patients with rheumatoid arthritis treated at the Rheumatology Clinic of RSCM during the period of April–May 2019 who met the inclusion criteria and did not meet the exclusion criteria. The sample was selected by consecutive sampling. The inclusion criteria were rheumatoid arthritis patients according to the 2010 ACR/EULAR diagnosis criteria, premenopausal women. Meanwhile, the exclusion criteria were taking steroids equivalent to prednisone > 7.5 mg per day for 3 months, patients with metabolic bone diseases that are known from medical records (such as hyperparathyroidism, Paget's disease, osteomalacia, and osteogenesis imperfecta), patients with other autoimmune diseases besides rheumatoid arthritis, taking drugs that affects the process of bone metabolism (such as bisphosphonates, hormonal therapy, antipsychotic drugs, antiseizure drugs, heparin, HCT, and furosemide), patients with chronic kidney disease with a glomerular filtration rate of <15 mL/min, patients with chronic liver diseases (liver cirrhosis and hepatoma), and patients with acute infectious diseases (fever and pneumonia). Laboratory tests in this study included TNF-*α*, SFRP-1, CTX, and P1NP examination using the ELISA method.

## 3. Results and Discussion

### 3.1. Results

This study involved 38 subjects that fulfilled the research criteria. The basic characteristics of the subjects in this study are shown in Table 1. For the proportion of glucocorticoid use thirty-one (81.6%) were noted to be treated with low-dose methylprednisolone < 4 mg, whereas seven were not on glucocorticoids at all. Thirty-one (81.6%) used MTX as their base combination of DMARD or as a monotherapy. For disease activity, twenty-three (60.6%) were in remission or low activity.

Based on disease activity subgroups, subjects were divided into two groups. The first group consisted of those with remission–low disease activity, and the second group consisted of those with moderate–high disease activity (Table 2).

**Figure 1:**
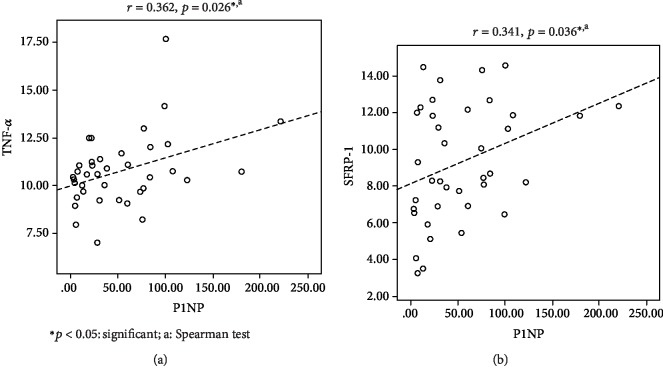
*Scatter plot* of correlation between TNF-*α* and P1NP (a); *scatter plot* of correlation between SFRP-1 and P1NP (b).

TNF-*α* level was positively correlated with P1NP (*r* = 0.362, *p* = 0.026, Figure 1(a)). And also, SFRP-1 level was positively correlated with P1NP (*r* = 0.341, *p* = 0.036, Figure 1(b)). However, TNF-*α* level did not correlate with SFRP-1 (*p* = 0.415) or CTX (*p* = 0.942).

## 4. Discussion

This study revealed that the median of the TNF-*α* level was 10.58 pg/mL. It is lower than the results of a study by Thilagar et al. [19] on RA patients in India, which found that the mean of the TNF-*α* level in RA patients was 17.9 pg/mL. However, the TNF-*α* level in this study was twofold higher than that of the normal control in their study, which was 5.5 pg/mL. The previous studies revealed the correlation between TNF-*α* level and disease activity [20, 21], so that the TNF-*α* level is low in remission or low disease activity. Besides, patients' inflammatory factors in this study are controlled by DMARD therapy, which could affect the TNF-*α* level [22]. In this study, the majority of subjects took methotrexate (MTX), which can inhibit cytokine production caused by T cell activation in RA patients [23]. In this study, other subjects also used a combination of DMARD therapy. The study of Osiri et al. [24], which also used a combination of 2 DMARD and 3 DMARD, showed low TNF-*α* level, 1.22 pg/mL and 1.44 pg/dL, respectively.

The mean of SFRP-1 levels in this study was 9.29 ng/mL. These levels were higher compared to the healthy population found in the previous study by Niu et al. [25] on nine patients, two men and seven women, which showed that the average of SFRP-1 levels in healthy control was 2.61 ng/mL. The previous study on SFRP-1 in RA was limited to synovial tissue of RA patients and gene expression in experimental animals. In several studies, an increase in SFRP-1 in inflammatory arthritis was found [12, 14, 15, 26]. The studies stated that the presence of cytokines in inflammatory arthritis conditions, such as RA, may induce synovial tissue, especially fibroblasts like synoviocyte, to express SFRP-1.

CTX levels that act as a marker of bone resorption activity in this study had a mean score of 2.74 ng/mL. It was higher than that of the previous study in Malang, which examined 47 RA patients, which was 0.59 ng/mL [27]. The reference value of normal CTX levels is below 0.3 ng/mL in healthy humans under 45 years old [28] and 0.2 ng/mL in healthy premenopausal women [29]. Compared with these results, CTX levels in this study were ten times higher than normal CTX levels. The majority of subjects in this study were in remission and low disease activity, but there was an increase in CTX levels, indicating that bone resorption activity continued even in controlled disease conditions.

P1NP levels which represent bone formation activity in this study had a median of 34.04 pg/mL or equivalent to 0.034 ng/mL. As a comparison, the average of P1NP level in the previous study on 64 healthy premenopausal and perimenopausal women was 37 ng/mL [30]. In the study of Fassio et al. [31], it was 39.19 ng/mL in 28 RA patients and 42.49 ng/mL in 35 healthy controls. It can be seen that the P1NP level in RA patients in this study was very low compared to that of healthy people.

In this study, there was no correlation between TNF-*α* and SFRP-1. This result may be caused by the fact that the subjects had low TNF-*α* levels, and the majority of subjects were in remission or low disease activity. However, the high SFRP-1 levels as a Wnt signaling inhibitor that may inhibit osteoblast differentiation and maturation in this study may be caused by induction from other proinflammatory cytokines. As shown in the study of Shaw et al. [32], in the case of inflammatory arthritis, IL-17A cytokines can also induce osteoblasts to express SFRP-1. The low levels of TNF-*α* and increased SFRP-1 levels in this study may indicate that after the RA process has progressed, molecularly, bone metabolism will continue to be disrupted resulting in the loss of bone mass even though the inflammatory factor has been suppressed. The previous study by Ideguchi et al. [33] assessed radiologically 122 AR patients that were treated with a conventional DMARD. There were 13 patients (10.7%) that showed improvement in bone erosion, but 4 out of the 13 patients still experienced new erosion. The study also found eight patients who experienced persistent remission, but no improvement in bone erosion, even three patients with persistent remission experienced radiological progression. The study concluded that the loss of bone mass in RA might continue even if the patient is in remission.

In this study, there was no correlation between TNF-*α* and CTX. On the contrary, the study in Malang that also examined premenopausal female patients with RA indicated that TNF-*α* levels had a significant correlation with CTX with a correlation coefficient of 0.615 [27]. However, the study did not assess P1NP levels. The absence of correlation between TNF-*α* and CTX may be caused by the low TNF-*α* levels in this study since the majority of patients were in remission and low disease activity and had received DMARD therapy. Besides, the possibility of induction of other proinflammatory cytokines not examined in this study may play a role in increasing CTX levels. As shown in the study of Chabaud et al. [34], IL-17 also increases bone resorption, and the presence of IL-1 in the study further increases the effects of IL-17. It can be concluded that the orchestra of several cytokines can strengthen the effects of osteoclast activity to carry out bone resorption in RA patients.

In this study, we found a weak positive correlation between TNF-*α* and P1NP. This result is in contradiction to the existing theory, which mentioned that the bone formation process would decrease if there is an increase in TNF-*α* levels. So far, TNF-*α* is known as an osteoblast inhibitor, and it increases osteoclastogenesis. However, the pathways and mechanisms activated by TNF-*α* involved in bone remodeling are still unclear. There are recent contradictory findings showing that TNF-*α* can also activate osteoblastogenesis. An in vitro study conducted by Huang et al. [35] found low MSC in mice that were given various TNF-*α* in different concentrations, around 0.001-0.1 ng/mL. TNF-*α* can induce an increase in the expression of Runx2 and Osterix needed for osteogenic differentiation. Another study that also used human mesenchymal stem cells (hMSC) was conducted by Hess et al. [36]. The study showed that TNF-*α* could trigger the osteogenic differentiation of hMSC by triggering NF-*κ*B, which will increase BMP-2 regulation and eventually cause an increase in the expression of Runx2 and Osterix. This study also found that the paradoxical role of TNF-*α* in bone homeostasis seems to depend on TNF concentration and differentiation state of the cell type used during the exposure. A novel cytokine IL-33 that play a role in the pathogenesis of many inflammatory diseases also has a pleiotropic effect, especially on bone remodeling; Ginaldi et al. [37] showed that IL-33 has a positive correlation with P1NP and a negative correlation with CTX, where the effects of cytokines on bone may vary depending on the stage of the disease and hormonal influences and the final effect of that cytokines on bone are therefore strongly conditioned by the clinical context. The existence of a weak positive correlation in this study may be caused by TNF-*α* levels that are quite low and the occurrence of bone metabolism so that there is a correlation with the P1NP marker. However, one resorption marker and one formation marker are required for bone turnover marker assessment. In this study, resorption is represented by CTX, and formation is represented by P1NP. Instead of being assessed individually, both markers have to be evaluated at once to get a complete picture of the direction of bone turnover [18, 38]. Perceived from the two markers in this study, turnover was more directed towards resorption activity, with an increase in CTX levels, which could not be balanced with P1NP levels.

In this study, we found a weak positive correlation between SFRP-1 and P1NP. Moreover, the findings in this study are very contradictory to the existing theory. As a Wnt signaling inhibitor, an increase in SFRP-1 will cause a decrease in bone formation activity characterized by decreased P1NP levels. However, current studies are still limited to experimental studies on animals, and no studies have used SFRP-1 serum in RA patients. However, physiologically, SFRP-1 is a secreted protein released by osteoblasts and osteocytes to maintain a balance of bone remodeling. An in vitro study by Bodine et al. [39] showed that the expression of SFRP-1 mRNA increased 24-fold during osteoblast differentiation, but overexpression of SFRP-1 in osteoblasts would accelerate the cell death rate by three times. The positive correlation of this study might be explained from this. There was a process of osteoblast activity in the case of bone formation which released various factors for growth followed by the production of various inhibitors, including SFRP-1, in some subjects experiencing remission and low activity. Thus, there is a possibility of an increase in SFRP-1 along with the increase in P1NP. The process of osteoblastogenesis may occur since the subjects in this study were premenopausal women. Estrogen plays a role in the endogenous response of osteoblasts, causing osteoblasts to differentiate and ultimately express type I collagen, bone matrix protein, various growth factors, and cytokines [40, 41]. Besides, the existence of other cytokines may increase SFRP-1 through osteoblasts as in the study of Shaw et al. [32], which found that IL-17A can induce the expression of SFRP-1 mRNA in osteoblasts. And also, a novel cytokine such as IL-31 that is involved both in AR and in bone-related diseases, as in the study of Ginaldi et al. [42], found that IL-31 in osteoporotic patients were significantly higher compared to controls. However, in this study, bone formation activity was minimal with low P1NP compared to bone resorption activity, which was indicated by CTX levels that were ten times of normal reference values. Therefore, it can be concluded that an imbalance of bone metabolism leading to resorption occurred at the time of measurement. It shows that the coupling process between osteoblasts and osteoclasts was disrupted since the high increase in resorption activity cannot be fully balanced by bone formation activity. This process will occur continuously, and it indicates the continuous loss of bone mass in RA.

Data from subgrouping analysis based on disease activity (Table 2) revealed that on the characteristics showed that patients with moderate–high disease activity have higher TNF-*α* and SFRP-1 values than those with remission–low disease activity, 11.05 pg/mL and 10.57 ng/mL, respectively. There was not much difference between the bone resorption marker (CTX) between the two groups, i.e., 2.77 ng/mL in the remission–low disease activity group and 2.70 ng/mL in the moderate–high disease activity group. However, both values are higher than the normal levels in previous studies [28], so that there was an increased resorption activity in both groups. For a bone formation marker (P1NP), it was higher in the remission–low disease activity group compared to the moderate–high disease activity group, i.e., 36.41 pg/mL and 31.10 pg/mL, respectively. It may indicate that bone formation process occurs in remission or low disease activity, but the process still cannot compensate for the existing resorption activity, so the turnover continues to run towards bone resorption, and over time it will cause erosion and loss of bone mass in RA patients.

The strength of this study is that this is the first study to simultaneously examine the correlation of inflammatory cytokines, in this case between TNF-*α* and Wnt signaling inhibitor, as well as SFRP-1 and resorption and bone formation markers, i.e., CTX and P1NP. This study is the first study to examine SFRP-1 using the serum as research material on RA patients, while the previous studies used synovial tissue [15]. This study also excluded chronic diseases or other factors that may cause the loss of bone mass, so that it was expected that the results could describe the loss of bone mass caused by RA.

The limitations of this study were the inability of this study to perceive the causal effect because the research design was cross-sectional. This study used the serum as the material that may be different from synovium, which inflammatory may be occurring only in the joints. Besides, this study did not examine other Wnt signaling inhibitors, such as DKK-1 and sclerostin. Therefore, the overall effect of Wnt inhibitors on bone metabolism could not be seen. In this study, the subjects were RA patients who had suffered RA quite a long time and had received therapy so that the effects of drugs on the process of bone metabolism could not be eliminated.

## 5. Conclusions

Premenopausal rheumatoid arthritis patients with remission and low disease activity have low levels of TNF-*α* proinflammatory cytokines, and it has no correlation with the high level of osteoblastogenesis inhibitor (SFRP-1) and the high level of bone resorption marker (CTX), but TNF-*α* and SFRP-1 levels have a weak positive correlation with the low bone formation marker (P1NP). Low level of TNF-*α* proinflammatory cytokines was found, but the high level of SFRP-1 and bone resorption marker (CTX) were not followed by bone formation activity as indicated by low levels of bone formation marker (P1NP), resulting in bone turnover imbalance.

## Figures and Tables

**Table 1:** Characteristics of subjects.

Characteristics	Result
Age (year), mean ± SB	38.82 ± 7.25
Duration of disease (year), median (min score–max score)	5 (1-19)
BMI, median (min score–max score)	21.82 (14.5–37)
Glucocorticoid dosage, *n* (%)	
(i) None	7 (18.4%)
(ii) Methylprednisolone < 4 mg	25 (65.8%)
(iii) Methylprednisolone 4 mg	6 (15.8%)
RA treatment, *n* (%)	
(i) Monotherapy MTX	21 (55.3%)
(ii) Combination of 2 DMARD	9 (23.7%)
(iii) Combination of 3 DMARD	1 (2.6%)
(iv) Monotherapy DMARD other than MTX	7 (18.4%)
CRP (mg/dL), median (min score–max score)	4.2 (0.6–88.4)
DAS28 CRP, *n* (%)	
(i) Remission	17 (44.8%)
(ii) Low	6 (15.8%)
(iii) Moderate	14 (36.8%)
(iv) High	1 (2.6%)
TNF-*α* (pg/mL), median (min score–max score)	10.58 (7–17.7)
SFRP-1 (ng/mL), mean ± SB	9.29 ± 3.17
CTX (ng/mL), mean ± SB	2.74 ± 1.37
P1NP (pg/mL), median (min score–max score)	34.04 (2.46–220.61)

**Table 2:** Characteristics of subject subgroups based on disease activity.

Characteristics	Remission–low disease activity (*n* = 23)	Moderate–high disease activity (*n* = 15)
CRP (mg/dL), median (min–max score)	3.0 (0.6–36.8)	7.2 (0.8–88.4)
TNF-*α* (pg/mL), median (min–max score)	10.27 (7.00–14.15)	11.05 (9.22-17.67)
SFRP-1 (ng/mL), mean ± SB	8.48 ± 3.24	10.57 ± 2.73
CTX (ng/mL), mean ± SB	2.77 ± 1.17	2.70 ± 1.69
P1NP (pg/mL), median (min–max score)	36.41 (3.46–220.61)	31.10 (4.90–179.24)

## Data Availability

The data used to support the findings of this study are available from the corresponding author upon request.
